# Is central venous catheter in haemodialysis still the main factor of mortality after hospitalization?

**DOI:** 10.1186/s12882-023-03433-6

**Published:** 2024-03-08

**Authors:** Erwin Campos, Miguel Angel Cuevas-Budhart, Renata Cedillo-Flores, Julián Candelario-López, Rigoberto Jiménez, Alberto Flores-Almonte, Alfonso Ramos-Sanchez, Jose C. Divino Filho

**Affiliations:** 1 Salvador B. Gautier Hospital, Santo Domingo, Dominican Republic; 2 Macrotech Dominican Republic, Santo Domingo, Dominican Republic; 3 Medical Research Unit in Nephrological Diseases of the Mexican Institute of Social Security, México City, México; 4 Iztacala Faculty of Higher Studies, National Autonomous University of Mexico, Mexico City, Mexico; 5 Medical Center for Diabetes, Obesity and Specialties, Santo Domingo, Dominican Republic; 6 Division of Renal Medicine, CLINTEC, Karolinska Institutet, Stockholm, Sweden

**Keywords:** Chronic Kidney Disease, Haemodialysis, Vascular Access, Mortality rate, Hospitalization rate, Comparison CVC versus AVF

## Abstract

**Background:**

Haemodialysis is the most frequently prescribed Renal Replacement Therapy modality worldwide. However, patients undergoing this therapy have an unpredictable evolution related to vascular access.

**Objective:**

To determine the factors associated with the mortality and hospitalization rate in haemodialysis patients at a third-level care Centre in the Dominican Republic.

**Methods:**

This was an observational and prospective study involving a cohort of 192 haemodialysis patients. The patient selection was non-probabilistic for convenience, and a direct source questionnaire was applied.

**Results:**

Of the 192 patients in the cohort, 103 (53.6%) were hospitalized and evaluated. The most frequent cause of hospitalization was catheter-related bloodstream infections (53.4%). Almost one-third (28.2%) of the hospitalized patients died, mostly due to infections (12.6%). Of those who died 29 patients (90%) had a Central venous catheter (CVC) with a non-tunnelled catheter (NTCVC) (65.5%); having an NTC CVC makes a patient 85.5 times more likely to be hospitalized than patients with arteriovenous fistulas.

**Conclusion:**

Vascular access plays a predominant role in the hospitalization and mortality rates in haemodialysis. Patients with an arteriovenous fistula obtained significantly better outcomes than those with central venous catheters.

## Introduction

Functioning vascular access is essential for performing haemodialysis. Morbidity and mortality in a haemodialysis program may be associated with the type of vascular access utilized at the beginning and during the follow-up of renal replacement therapy (RRT) [1–4]. The types of vascular access (VA) include Autologous Arterial Venous Fistula (AVF), Arteriovenous Graft (AVG), and Central Venous Catheter (CVC), which can be tunnelled (T-CVC) or non-tunnelled (NT-CVC). While haemodialysis may present different complications during the sessions, complications associated with VA occur in 16–25% [5–7]. Some reports suggest that CVC is associated with a higher mortality risk than AVF and AVG. It is worth mentioning that the survival of patients with stage 5 CKD has improved considerably over the years [8–10].

The aim of the study is to determine the factors associated with mortality, hospitalization, and mortality after hospitalization in haemodialysis patients at the Salvador Bienvenido Gautier Hospital in Santo Domingo, Dominican Republic.

## Methodology

A prospective observational study of 192 patients undergoing HD treatment at Salvador Bienvenido Gautier Hospital in Santo Domingo, Dominican Republic, was conducted. The Renal and Safety Committee of Gautier Hospital and the National Health System (SNS) approved the study. The sample was non-probabilistic for convenience and included all patients of both sexes who were 18 years of age or older, receiving treatment for at least three months at the time of selection with any type of VA, and who had been hospitalized during the study period, which was from December 2018 to July 2019.

A data collection instrument was designed and drafted; it included sociodemographic data, type of VA and duration of use, viral infections, date of admission to haemodialysis, comorbidities, hospitalization dates, outcomes, and cause of mortality. The patients accepted and signed the informed consent in which the confidentiality of the results was guaranteed. Data was collected by direct interrogation of the patient and by reviewing their clinical chart.

### Statistical analysis

The dependent variables were mortality and hospitalization, whereas the independent variables were age, gender, type of VA, comorbidities, reason for hospital admission, patient discharge condition and cause of death.

The first analysis involved exploring the data using descriptive statistics. Simple frequencies and percentages were described to qualitative variables, while mean and standard deviation (X ± SD) were used to describe quantitative variables. Temporal trends were analysed by evaluating mortality and hospitalization rates.

In the second analysis, inferential statistics were used to determine the relationship between hospitalization and patient mortality. Binary logistic regression analysis was used with a 95% confidence interval.

Finally, a third multivariate analysis was performed using multiple logistic regression. This involved using variables that were significant in the bivariate analysis. The regression model was then adjusted using the Hosmer-Lemeshow goodness test as an indicator of fit. This is to identify factors that best explain the association with mortality and hospitalization. Derived from the model, association measures were estimated using OR with a 95% confidence interval, and statistical significance *p*-value < 0.05.

The information obtained, was organized and processed using the statistical program SPSS version 25 (IBM Corp. Armonk, N.Y., USA).

## Results

A total of 192 patients who met the inclusion criteria were included. The mean age was 42.4 + 11.2 years. The majority of patients (38, 71%) were male with 77 patients (40%) having an AVF and 115 (60%) a CVC. Regarding the comorbidities, hypertension was the most frequent pathology in 183 patients (95%), followed by diabetes mellitus with 83 patients (43%), whereas 78 patients (41%) had both of these comorbidities. (Table 1 shows the data segmented by hospitalized patients).

**Table 1:** Sociodemographic characteristics and ethology of the disease

	Hospitalized		No hospitalized	
	**f**	**%**	**f**	**%**
Gender				
Male	73	71	65	73
Female	30	29	24	27
Age				
18–29	7	6.8	5	5.6
30–39	18	17.5	14	15.7
40–49	28	27.2	22	24.7
50–59	29	28.2	26	29.2
60 o más	21	20.4	22	24.7
Diabetes				
Yes	49	47.6	34	38.2
No	54	52.4	55	61.8
Hypertension				
Yes	98	95.1	85	95.5
No	5	4.9	4	4.5

During the follow-up period, 103 patients (54%) were hospitalized, of which 16 (15%) had AVF and 87 (85%) CVC; regarding mortality, 45 patients died (23%) (see Table 2). The principal cause of hospitalization was HD catheter-related bloodstream infections (CRBSI) which were present in 55 patients (53%) (24 NT-CVC; 31 T-CVC), followed by cardiovascular causes in 14 patients (14%), followed by infections (pneumonia, diabetic foot, urinary tract infection (UTI)), in 12 patients (12%). It is important to emphasize that four patients had infective endocarditis (IE) (4%) and three of them had an NT-CVC that was related to the IE and the catheter was repositioned to a femoral site and given 4–6 weeks of antibiotic. The outcome of IE consisted of one deceased and three recovered, the survival ones remained on HD. (See Table 3.)

**Table 2:** Hospitalization and Mortality

	No hospitalizations n (%)	Hospitalizations n (%)	Hospitalized no death (%)	Hospitalized + death n (%)
AVF	61 (68.5)	16 (15.5)	69 (46.9)	8 (17.8)
T-CVC	26 (29.2)	40 (38.8)	50 (34.0)	16 (35.6)
NT-CVC	2 (2.2)	47 (45.6)	28 (19.0)	21 (46.7)
Total of patients 192	89 (46.3)	103 (53.7)	147 (76.6)	45 (23.4)

Source: Direct (AVF: Autologous arteriovenous fistulas, T-CVC: tunneled catheters NT-CVC: non-tunneled catheters)

**Table 3:** According to the Ethology of Hospital Admission

Cause of Hospitalization	NT-CVC	T-CVC	AVF	Total
	n (%)	n (%)	n (%)	n (%)
CRBSI	24 (51.1)	31 (77.5)	0 (0)	55 (53.4)
Uremic Syndrome	1 (2.1)	0 (0)	1 (6.3)	2 (1.9)
Fluid Overload	0 (0)	0 (0)	2 (12.5)	2 (1.9)
Infective Endocarditis	3 (6.4)	0 (0)	1 (6.3)	4 (3.9)
Vascular Access Dysfunction	1 (2.1)	3 (7.5)	1 (6.3)	5 (4.9)
Hematologic, Severe Anaemia	1 (2.1)	1 (2.5)	1 (6.3)	3 (2.9)
Infectious (Pneumonia*, Diabetic Foot, UTI)	7 (14.9)	3 (7.5)	2 (12.5)	12 (11.7)
Cardiovascular (AMI, CVD)	5 (10.6)	2 (5)	7 (43.8)	14 (13.6)
Gastrointestinal bleeding	5 (10.6)	0 (0)	1 (6.3)	6 (5.8)
**General Ethology**	**47 (45.6)**	**40 (38.8)**	**16 (15.6)**	**103(100)**

Source: Direct (AVF: Autologous arteriovenous fistulas, T-CVC: tunneled catheters NT-CVC: non-tunneled catheters, CRBSI: Catheter-related bloodstream infections, AMI: acute myocardial infarction: CVD: cardiovascular disease, UTI: urinary tract infection). * Pneumonia cases are superimposed pulmonary infections unrelated to vascular access

Starting from the bivariate analysis between the variables of interest (sociodemographic, comorbidities, type of VA) and the dependent variables (hospitalization and mortality), it was shown that there is a statistical significance (*p* < 0.05). The significant variables were the type of VA (*p* < 0.001) and more than one comorbidity (*p* < 0.037).

Regarding hospitalization, it was found that patients having NT-CVC (OR: 85.5; 95% CI: 19.62-409.99) are 85.5 times more likely to be hospitalized and patients having T-CVC (OR: 5.86; 95% CI: 2.80-12.28) only six times more when they are compared to patients with AVF. The mortality rate for HD patients was 43.2 deaths /100 patient-years.

Finally, in the multivariate analysis, taking death after hospital admission as the dependent variable, it was found that having a NT- CVC (OR: 6.58; 95% CI: 2.58–16.78) and T-CVC (OR: 2, 92; 95% CI: 1.14–7.43) and having more than two comorbidities, (OR: 2.04; 95% CI: 1.01–4.15), compose the strongest association with death. In other words, in the probability analysis, we found that the patient who has CVC (NT-CVC or T-CVC) and who also has more than two comorbidities (arterial hypertension and diabetes mellitus) is 6.97 times more likely to die after hospitalization.

## Discussion

This study shows that having a high mortality rate after hospitalization is a phenomenon that occurs in the entire population undergoing HD treatment.

Regarding VA, the patients who were most hospitalized for any cause had an NT-CVC (45.6%), followed by those who had a T-CVC with 38.8%. It shows that nearly all the hospitalized HD population has CVC (84.4%). Lacson E. et al. [11] have reported results of patients with CVC between 39 and 45%, which may be very similar in terms of results to ours; however, in their report, the patients migrate to AVF promptly.

One of the reasons CVC patients are hospitalized more frequently than AVF is that they may be more prone to infection and malfunction. In addition, most dialysis patients in the Dominican Republic debut to HD as an emergency without prior AVF access, so they start with an NT-CVC and then change it to a T-CVC while the AVF is performed. This change of type of catheter (NT-CVC to T-CVC) is generally borne by the patient, which often prevents it from being carried out promptly.

Most guidelines suggest a goal of around 80% AVF in patients with chronic HD [12]. The prevalence of AVF in the study period in our HD universe was 40.1%, whereas CVC was 59.9% (34.4% T-CVC and NT-CVC 25.5%). In the Dominican Republic, the number of vascular surgeons is limited, as a result, the waiting list for fistulas can be extended for a period of more than three months, which means that many of the patients continue with CVC.

The high prevalence of catheters, plus their high risk of hospitalization, means that Hospital Salvador Bienvenido Gautier in the Dominican Republic has a higher incidence of hospitalizations, potentiated by the high probability of infection and dysfunction.

Hypertension is more commonly observed in a dialysis patient, but this does not necessarily mean that it is the aetiology of stage 5 CKD. The reason for having a higher prevalence of uncontrolled hypertension in HD may be due to the not-successful treatment of fluid overload or not being able to reach the dialysis “Dry Weight.” In addition, another possible reason for high blood pressure is poor adherence to antihypertensive treatment patients may have, probably cost-related, as most patients live at or near the poverty line.

Compared with Agarwal R. et al. [13], the percentage of hospitalized patients with hypertension was higher at a 95.1%, while they reported 86%. Referring to a very varied range in patients with arterial hypertension that goes from 50 to 86%, this does not differ from the present results or those found by Amber O. Molnar et al. [14], who reported that 76.5% of the patients were hypertensive.

The main reasons for hospitalization in the present study were CRBSI, which is one of the most frequent, lethal, and costly complications of central venous catheterization [13] (53.4%), followed by cardiovascular causes (AMI, CVD) (13.6%), and other infections (Pneumonia, Diabetic Foot, UTI) (11.7%). When comparing it with the work of Pantoja A et al. [17], which showed CRBSI as the first cause (38.5%) and second, Pneumonia (28.2%), whereas Polanco del Orbe et al. [18] had CRBSI as first reason in 56% of the cases.

A high percentage of hospitalizations associated to catheter infections is expected, due to the high incidence of CVC in the Dominican Republic, which is the reason why CRBSI is more common in NT-CVCs; also, these infections types are the most frequent cause that forces the withdrawal of any access [16]. At this point, we are faced with the use of good evidence-based practices for all patients, as mentioned by Craswell et al. [19]. Therefore, creating a tunnel for the catheter may prevent bacterial translocation from the skin to the bloodstream; however, this does not prevent bacteraemia, due to poor access management of the catheter during the connection and disconnection processes of a HD session.

Regarding the mortality found in this study (23.4%), the data are in agreement with those (27.3%) found by Ahmed, M. et al. [13] in a study conducted in Dubai. In Ahmed et al. study, the most compelling cause of death was cardiovascular (42.8%), followed by Infection/sepsis (18.7%). This differs from the cause of mortality in the present study, in which infection is primarily found, followed by cardiovascular disease. This may be due to better VA control in Middle Eastern patients.

One of the issues observed with the catheters in the study is that a moderate percentage (28.2%) of hospitalized patients died, Additionally, catheters can impact the efficiency of HD therapy by reducing the blood flow during haemodialysis.

Finally, Giraldo Y. et al. [20]. in Spain reported in their multivariate analysis that the vascular catheter, as an independent variable, is a predictor of mortality during hospitalization, as shown in this study. This may indicate that regardless of the idiosyncrasy, geographical area, type of patient, socioeconomic level, infections, and comorbidities are predisposing factors for the deterioration of the patient in HD therapy.

### Implications for health policies

The results of this study reflect the reality of a public hospital in the Dominican Republic and highlight the importance of creating health policies that promote the development of AVFs, as it ensures more remarkable patient survival and reduces hospitalizations and associated costs.

The use of NT-CVC should be avoided since it is an independent predictor of mortality in hospitalized HD patients, undoubtedly improving the standards of quality and care in patients being treated with HD at the hospitals of the National Health Service of the Dominican Republic.

### Limitations of the study

From the results indicated here, it is important to mention that the patient population on HD had a high risk of developing endocarditis; however, at the time of the study, not all patients had echocardiography performed due to the cost of echocardiography at that time, a significant expense for an underdeveloped country., In addition, the Charlson Comorbidity index was not available, which leaves it open to future research that includes these variables.

There were not enough variables to fit the predictive models at the same time. However, the association between catheter use, comorbidity, and bacteria are responsible for haemodialysis association infections, and hospitalizations is strong enough to confirm our hypothesis. This reinforces the need to have a programmed early VA program, which consists of guarantees a more adequate and secure VA for the patient.

## Conclusion

Our results suggest that patients with CVC show a higher percentage of hospitalization and mortality rates when compared to patients with AVF. More than half of hospitalizations are due to CRBSI (which is associated with a higher mortality rate), followed by cardiovascular causes.

Furthermore, NT-CVC patients play a predominant role in the rate of hospital admission and mortality, especially if they have more than one comorbidity, as they have a high probability of dying. In contrast, patients with AVF achieve better outcomes compared to CVC users.

These findings reinforce the need having to have a pre-dialysis VA protocol to ensure that patients are ready, with an appropriate VA (either AVF or AVG) when it is time to start haemodialysis.

## Data Availability

In relation to the availability of the data, the contact that will be available to provide the required data is the correspondence: Miguel Angel Cuevas-Budhart. Associate Researcher “B”. Coordinator of Nursing programs of the Medical Research Unit in Nephrological Diseases of the Mexican Institute of Social Security. Email: angel_budhart@hotmail.com, angel.budhart@gmail.com.
